# Traditional Social Sports Games and Mental Training for Smartphone Addiction and Psychological Distress in School-Aged Adolescents: Randomized Controlled Trial

**DOI:** 10.2196/85353

**Published:** 2026-05-04

**Authors:** Mohamed Yaakoubi, Ahmed Ghorbel, Hiba Abdelkafi, Liwa Masmoudi, Omar Trabelsi, Safaa M Elkholi, Fatma H Yagin, Georgian Badicu, Adnene Gharbi

**Affiliations:** 1 High Institute of Sports and Physical Education of Kef, University of Jendouba, Kef, Tunisia; 2 Research Unit: Physical Activity, Sport and Health, UR18JS01, National Observatory of Sport, Tunis, Tunisia; 3 The High Institute of Applied Studies in Humanities of Mahdia, Department of Education Sciences, University of Monastir, Mahdia, Tunis, Tunisia; 4 Department of Rehabilitation Sciences, College of Health and Rehabilitation Sciences, Princess Nourah bint Abdulrahman University, P.O. Box 84428, Riyadh 11671, Riyadh, 84428, Saudi Arabia,; 5 Department of Biostatistics, Faculty of Medicine, Malatya Turgut Ozal University, 44210, Malatya, Turkey; 6 Department of Physical Education and Special Motricity, University Transilvania of Braşov, Braşov, Romania; 7 High Institute of Sports and Physical Education of Sfax, University of Sfax, Sfax, Tunisia

**Keywords:** smartphone addiction, adolescents, loneliness, psychological distress, randomized controlled trial, Tunisia

## Abstract

**Background:**

Problematic smartphone use among adolescents is a growing public health concern closely associated with psychological distress and loneliness. Effective, culturally grounded, school-based interventions are needed.

**Objective:**

The aim of this study was to assess the effects of a 12-week program combining traditional social sports games and mental exercises on smartphone addiction, nomophobia, psychological distress, and loneliness in adolescents.

**Methods:**

In this randomized controlled trial, 69 school-recruited Tunisian adolescents (aged 14-16 years) with clinically elevated smartphone addiction scores were assigned to an experimental group (n=36, 52.2%) or a control group (n=33, 47.8%). The experimental group received a 12-week intervention comprising 4 weekly sessions integrating traditional social sports games with mental exercises, whereas the control group continued standard physical education. Outcomes (smartphone addiction, nomophobia, psychological distress, and loneliness) were assessed at baseline and after the intervention using scales validated in Arabic.

**Results:**

Linear mixed-effects models adjusted for age, sex, and BMI revealed significant group × time interactions of moderate magnitude across all outcomes (*P*<.05 in all cases) favoring the experimental group. Adjusted postintervention comparisons confirmed significantly lower scores in the experimental group for smartphone addiction, nomophobia, psychological distress, and loneliness (*P*<.05 in all cases; partial η_p_^2^=0.08‐0.12). Mediation analysis indicated that reductions in loneliness accounted for 34.4% of the intervention’s effect on smartphone addiction, consistent with partial mediation.

**Conclusions:**

A culturally adapted, school-based intervention combining traditional social sports games and mental exercises significantly reduced problematic smartphone use and improved psychological well-being. The partial mediation through reduced loneliness highlights the critical role of social connectedness in adolescent digital health interventions.

## Introduction

Adolescence constitutes a critical period of neurobiological, emotional, and social development marked by heightened sensitivity to reward processing, peer feedback, and affective stimuli. Ongoing maturation of prefrontal-limbic circuits during this stage increases impulsivity and emotional reactivity, rendering adolescents particularly vulnerable to maladaptive behavioral patterns in technology-saturated environments [1].

In Tunisia, these developmental vulnerabilities intersect with rapid digital expansion that has outpaced public health policy and digital literacy initiatives. As a result, smartphones have become central tools for managing academic demands, emotional regulation, and social relationships among adolescents [2-4]. While digital access offers clear benefits, excessive reliance on digital devices has contributed to the growing prevalence of problematic smartphone use (PSU).

PSU is characterized by compulsive, excessive smartphone engagement that results in functional impairment across academic, emotional, and social domains [5]. Among its core manifestations, nomophobia, the fear of being without one’s smartphone, has emerged as a prominent indicator of psychological dependence and psychological distress [6,7]. Empirical evidence consistently links PSU and nomophobia to emotional dysregulation; reduced academic performance; and elevated symptoms of anxiety, depression, and stress [8].

Loneliness is a key psychosocial mechanism underlying PSU. Adolescents often engage in smartphone use to compensate for perceived social disconnection, yet digitally mediated interactions frequently fail to satisfy deeper relational needs. This mismatch can create a self-reinforcing cycle where increased smartphone use exacerbates loneliness, further driving compensatory digital engagement [9,10]. Therefore, interventions that disrupt this cycle by directly enhancing social connectedness are critical. Meta-analytic evidence indicates that physical activity (PA) interventions, particularly those that are group based and sustained (≥12 weeks), effectively reduce smartphone addiction [11], with loneliness established as a mediator of this effect [12,13]. This process aligns with self-determination theory (SDT) as socially engaging PA can satisfy core psychological needs such as relatedness, thereby reducing reliance on smartphones for compensatory fulfillment [14].

SDT further explicates this mechanism, positing that psychological well-being depends on the satisfaction of 3 basic psychological needs: autonomy, competence, and relatedness [15]. From this perspective, PSU may represent a maladaptive coping strategy that emerges when the need for relatedness remains unmet. Thus, loneliness functions both as an indicator of need frustration and as a mechanistic pathway sustaining compulsive smartphone use.

Building on this rationale, PA interventions, particularly structured, group-based programs, have demonstrated efficacy in reducing PSU and improving psychological well-being among adolescents [16,17]. There is further evidence suggesting that PA-based interventions are more effective when combined with psychological strategies such as mental skill training, which strengthens emotional regulation, stress tolerance, and self-regulation capacities [11].

Cultural adaptation represents a critical determinant of engagement and sustainability. In the Tunisian context, traditional social sports games (eg, seated ball game and dodgeball) constitute culturally resonant, rule-based PAs that promote face-to-face interaction, cooperation, and prosocial behavior. These activities foster peer bonding and shared enjoyment, directly targeting the SDT need for relatedness while counteracting the isolating effects of excessive screen use [18,19].

Despite this strong theoretical and empirical rationale, important gaps remain. Few controlled studies have evaluated multi-component, culturally grounded interventions that integrate structured PA with psychological skill development using a randomized controlled design in a school setting. Moreover, no study has empirically tested whether reductions in loneliness mediate the effects of such interventions on PSU despite the central role of relatedness within SDT.

To address these gaps, we developed and evaluated a 12-week school-based intervention integrating traditional social sports games with mental exercises (TSSG-ME) among Tunisian adolescents. We hypothesized that participants in the TSSG-ME group would demonstrate significantly greater reductions in smartphone addiction and psychological distress than a control group (CG). Grounded in SDT, we further hypothesized that reductions in loneliness would mediate the intervention’s effect on smartphone addiction, reflecting enhanced satisfaction of the need for relatedness.

## Methods

### Study Design and Setting

This study was a 2-arm, parallel-group randomized controlled trial. The trial was retrospectively registered with the Pan African Clinical Trials Registry under the identification number PACTR202601838702413 on January 30, 2026. The registration was conducted retrospectively due to an administrative oversight during the initial recruitment phase as the research team prioritized data collection commencement following funding approval. This study was conducted between October 2024 and February 2025 across 3 public middle schools in the Sfax region of Tunisia during routine health screenings.

### Participants

A total of 960 adolescents (aged 14-16 years) were initially recruited during routine health screenings across 3 public middle schools in the Sfax region. A total of 151 students with PSU were identified. The inclusion criteria were (1) meeting the clinical cutoff for smartphone addiction and (2) reporting structured PA not exceeding 3000 metabolic equivalent of task minutes per week as assessed using the Arabic-language International Physical Activity Questionnaire [20]. The exclusion criteria were (1) BMI below the 3rd or above the 97th percentile [21] and (2) habitual sleep duration outside the range of 6 to 10 hours per night [22]. This resulted in the exclusion of 76 students (n=37, 48.7% due to excessive activity; n=39, 51.3% due to the BMI and sleep criteria).

Eligible participants (n=75) were randomly assigned to the experimental group (EG; n=36) or the CG (n=33) using stratified block randomization. Randomization was stratified by sex (male and female), with a combination of random block sizes of 4, 6, and 8 to ensure balanced allocation across groups throughout the recruitment period. The allocation sequence was obtained using computer-generated random numbers by an independent researcher who was not involved in participant recruitment, intervention delivery, or outcome assessment.

Allocation concealment was ensured using sequentially numbered opaque, sealed envelopes prepared by the independent researcher and opened only after completion of baseline assessments and confirmation of eligibility. Participants were enrolled by the lead author, and group assignment was implemented by a research assistant not involved in outcome assessment.

Due to the nature of the behavioral intervention, participants and intervention providers could not be blinded. However, outcome assessors and data analysts were blinded to group allocation throughout the trial.

**Figure 1.**
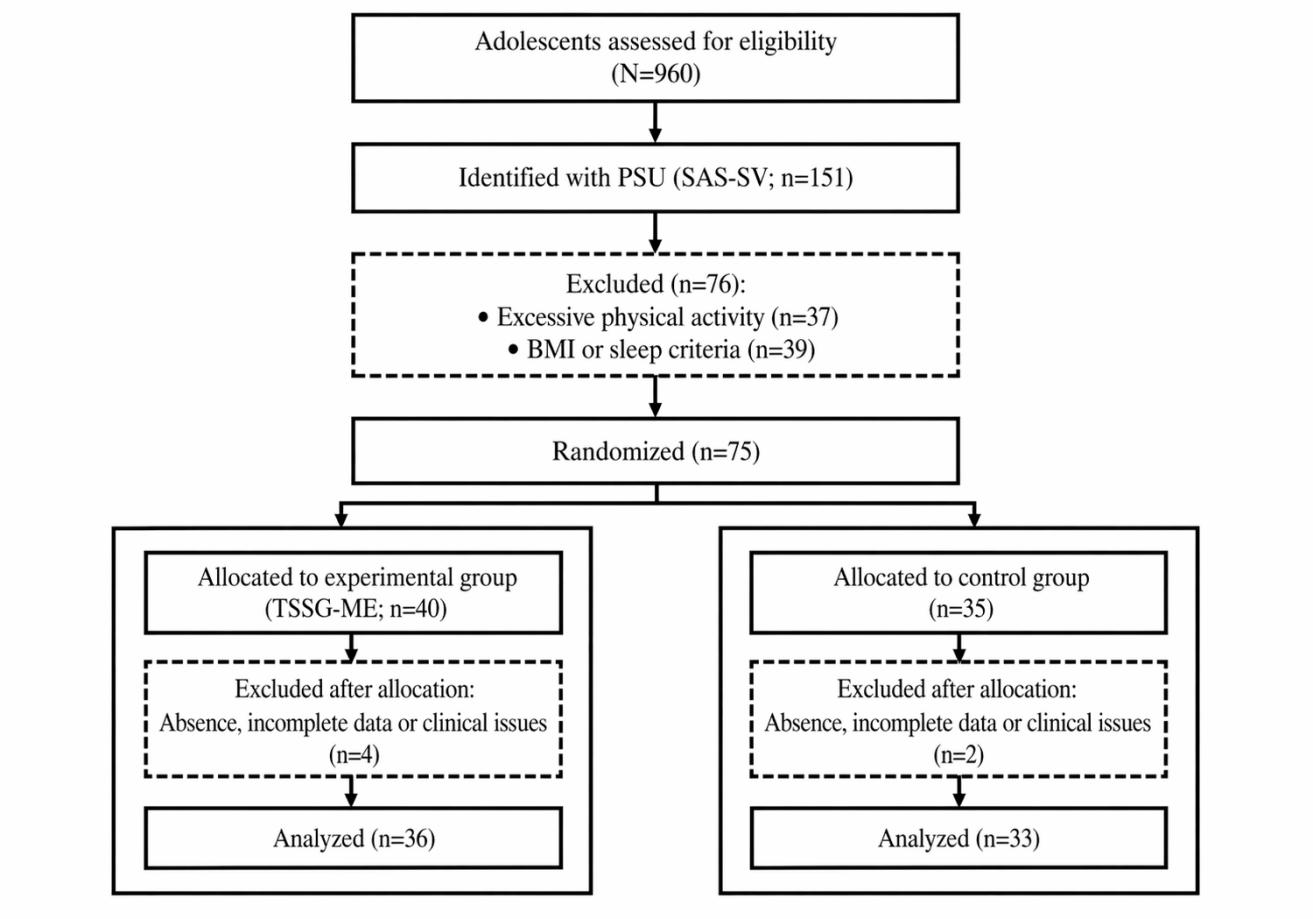
Participants’ flow through the phases of the randomized controlled trial. PSU: problematic smartphone use; SAS-SV: Smartphone Addiction Scale–Short Version; TSSG-ME: traditional social sports games with mental exercises.

During the trial, 8% (6/75) of the participants were excluded due to extended absences (>1 week), incomplete assessments, or newly emerging clinical conditions (EG: n=4; CG: n=2), resulting in a final analytic sample of 69 participants (EG: n=36, 52.2%; CG: n=33, 47.8%). The Little test was nonsignificant (*χ*^2^_18_=12.5; *P*=.82), supporting the assumption that data were missing completely at random. Given the low rate of missingness and the missing completely at random assumption, we conducted complete-case analyses without multiple imputation. Baseline comparisons confirmed equivalence between groups on all demographic and outcome variables (*P*>.05 in all cases). A detailed flowchart of participant screening, randomization, allocation, and follow-up is presented in Figure 1.

### Intervention

#### Overview

Grounded in SDT, the intervention was designed to target basic psychological needs. The traditional social sports game component primarily addressed the need for relatedness through cooperative and socially structured play, whereas the mental exercise component targeted competence and autonomy through structured skill building and self-regulation practices. The intervention began within 1 week following randomization for both groups.

#### Experimental Group

The EG participated in a structured 12-week intervention consisting of four 60-minute sessions per week. Three sessions were conducted during regular school hours, and 1 extracurricular session was held on Friday afternoons [23]. Each session was divided into 2 core modules: traditional social sports games (45 minutes) and mental exercises (15 minutes). The sports module used 4 culturally embedded Tunisian games (seated ball game, dodgeball, sbeïla [a traditional chasing game], and korra teyara [a traditional target throwing game]) implemented with an emphasis on teamwork, communication, and strategic decision-making [18]. Game complexity increased over time: weeks 1 to 3 focused on rule acquisition and teamwork, whereas weeks 4 to 12 introduced dynamic variations and collaborative problem-solving strategies [24,25].

The mental exercise module followed a standardized 5-phase sequence to facilitate cognitive and physical recovery [26,27]. This sequence began with a 2-minute prerelaxation phase for session orientation followed by a 3-minute induction to calmness using diaphragmatic breathing and guided imagery. The main relaxation phase (5 minutes) incorporated Jacobson’s progressive muscle relaxation, autogenic training, and mindfulness-based techniques. The session concluded with a 2-minute recovery period of light stretching and a 3-minute reflective closure involving verbal discussion or a short written reflection.

#### Control Group

The CG followed the standard, time-matched physical education curriculum mandated by the Tunisian Ministry of Education, consisting of 2 weekly sessions (one 2-hour and one 1-hour session) [23]. Sessions included conventional individual activities and team sports. The CG did not receive the novel social games or mental exercises, serving as a treatment-as-usual control. This design allowed for a pragmatic comparison with standard school practices but did not control for nonspecific intervention effects such as increased attention, novelty, or social interaction.

#### Instruction and Fidelity

Sessions were delivered by a team of 3 instructors, each with over 10 years of experience working with youth. The team comprised 2 physical education specialists with master’s degrees in adapted physical activity and sport pedagogy and 1 certified mental preparation coach with a professional certificate in mental skill training for adolescents. To ensure treatment fidelity, all instructors received comprehensive training on the research protocol and adhered to a standardized intervention manual. For each session, participants were organized into 3 subgroups, enhancing peer interaction and enabling close instructor supervision. Attendance was monitored via session registers, with the EG maintaining an average attendance rate of 97.7% (SD 2.4%).

### Data Collection

#### Procedures

The primary outcome of this study was smartphone addiction, measured using the SAS-SV. Secondary outcomes included nomophobia (Nomophobia Questionnaire; NMP-Q), psychological distress (Depression, Anxiety, and Stress Scale–21 [DASS-21] subscales), and loneliness (University of California, Los Angeles, Loneliness Scale). Trained assessors administered all instruments in Arabic during face-to-face sessions, with data collected electronically via Google Forms on digital tablets to minimize entry errors [28]. All assessments were conducted at 2 time points: baseline and immediately after the intervention.

#### Investigation Tools

##### Smartphone Addiction

Smartphone addiction was assessed using the SAS-SV, a self-report instrument designed to evaluate the severity of PSU and originally validated by Kwon et al [29]. The SAS-SV is a 10-item instrument rated on a 6-point scale (1=“strongly disagree”; 6=“strongly agree”), capturing dimensions such as daily life disturbance, withdrawal, cyberspace-oriented relationships, overuse, and tolerance. Sex-specific cutoff scores were used to classify participants as smartphone addicted (≥33 for girls and ≥31 for boys). Total scores range from 10 to 60, with higher scores indicating greater levels of PSU. An Arabic-adapted version of the SAS-SV**,** validated by Sfendla et al [30], was used to ensure linguistic and cultural appropriateness. In this sample, the scale demonstrated good internal consistency (Cronbach α=0.84).

##### Nomophobia

Nomophobia was assessed using the Arabic version of the NMP-Q, validated by Farchakh et al [31]. The 20-item instrument evaluates fear of being without a smartphone across four domains: (1) inability to communicate, (2) losing connectedness, (3) inability to access information, and (4) giving up convenience. Participants rated each item on a 7-point Likert scale (1=“totally disagree”; 7=“totally agree”). Total scores range from 20 to 140, with severity levels classified as absent (20), mild (21-59), moderate (60-99), and severe (100-140). The Arabic NMP-Q showed excellent internal consistency (Cronbach α=0.948) in this sample.

##### Depression, Anxiety, and Stress

Emotional distress was measured using the Arabic version of the DASS-21 validated by Ali et al [32]. The DASS-21 includes three 7-item subscales that independently measure depression, anxiety, and stress. Items are rated on a 4-point Likert scale (0=“did not apply to me at all”; 3=“applied to me very much/most of the time”). Severity was classified per subscale (eg, depression: 0‐9=normal; ≥28=extremely severe). The tool showed strong internal consistency for this age group (Cronbach α≥0.83).

##### Loneliness

Loneliness was assessed using the Arabic-adapted University of California, Los Angeles, Loneliness Scale–Short Form, validated by Alateeq et al [33]. This 8-item self-report tool evaluates perceived social isolation through statements such as “I don’t have a friend.” Responses are rated on a 4-point Likert scale (1=“never”; 4=“always”), with total scores ranging from 8 to 32. Higher scores indicate greater loneliness. Internal reliability was acceptable (Cronbach α=0.777), confirming suitability for adolescents in Tunisia.

### Sample Size

An a priori power analysis was conducted using G*Power (version 3.1) [34] for a repeated-measure ANOVA with a within-between interaction. To detect a medium effect size (η_p_^2^=0.06, equivalent to *f*=0.25) for the primary group × time interaction, with α of .05 and 1 − β of 0.80, a total of 64 participants were required; the final sample comprised 69 adolescents. The choice of a medium effect size is consistent with prior intervention research on adolescent digital addiction and related school-based programs, which reports moderate intervention effects on problematic digital technology use outcomes [35].

### Data Analysis

Data were analyzed using SPSS Statistics (version 28.0; IBM Corp). Baseline group differences were examined using independent 2-tailed *t* tests for continuous variables and chi-square tests for categorical variables. Primary intervention effects were evaluated using linear mixed-effects models, which included group, time, and the group × time interaction as fixed effects, with participants as a random effect. These models were adjusted for the covariates of age, sex, and BMI and tested for each outcome. Exploratory within-group pretest-posttest changes were assessed using paired *t* tests, and between-group differences at the posttest time point were examined using independent *t* tests. Analyses of covariance were conducted to compare adjusted postintervention outcomes between groups controlling for baseline scores as well as age, sex, and BMI. Effect sizes were calculated using η_p_^2^ and interpreted according to conventional benchmarks [36]. Mediation analyses were performed using bootstrapping procedures with 5000 resamples to estimate indirect effects and 95% CIs [37]. Statistical significance was set at an α value of .05 (2 tailed).

### Ethical Considerations

This study received ethics approval from the local Committee for the Protection of Persons at the Faculty of Medicine of University of Sfax, Tunisia (approval 0554/2023). The research was conducted in full compliance with the ethical standards outlined in the Declaration of Helsinki (2013). Written informed consent was obtained from participants’ legal guardians, and verbal assent was obtained from each adolescent prior to participation. All data, including smartphone use records, questionnaire responses, and behavioral observations, were anonymized. Any potentially identifiable information was stored separately on a password-protected server accessible only to the principal investigator. No financial or material compensation was provided to participants.

## Results

### Baseline Characteristics

A detailed flowchart of participant screening, randomization, allocation, and follow-up is presented in Figure 1. At baseline, no statistically significant differences were observed between the EG (n=36) and CG (n=33) across demographic, anthropometric, or psychological variables (Table 1). The groups were comparable in age, sex distribution, BMI, smartphone addiction, nomophobia, psychological distress (global and subscale scores), and loneliness (*P*>.05 in all cases). Effect size estimates were negligible, indicating satisfactory baseline equivalence prior to the intervention.

**Table 1.** Baseline characteristics of the participants (N=69)^a^.

Characteristics	EG^b^ (n=36)	CG^c^ (n=33)
Age (y), mean (SD)	15.0 (0.6)	15.1 (0.7)
Sex (female), n (%)	16 (44.4)	14 (42.4)
BMI (kg/m^2^), mean (SD)	20.7 (1.5)	20.6 (1.6)
SAS-SV^d^ score (10-60), mean (SD)	38.9 (3.0)	39.0 (3.2)
NMP-Q^e^ score (20-140), mean (SD)	105.2 (10.5)	106.0 (11.2)
DASS-21^f^ global score (0-126), mean (SD)	70.1 (15.0)	70.8 (16.5)
DASS-21 subscale scores, mean (SD)		
Depression (0-42)	28.2 (6.5)	28.5 (7.0)
Anxiety (0-42)	22.1 (5.0)	22.3 (5.5)
Stress (0-42)	19.8 (6.0)	20.0 (6.5)
ULS-8^g^ score (8-32), mean (SD)	26.8 (3.5)	27.0 (3.8)

a Independent *t* tests and chi-square tests confirmed no significant differences between groups at baseline. The variables of age, sex, and BMI were included as covariates in subsequent inferential models.

b EG: experimental group.

c CG: control group.

d SAS-SV: Smartphone Addiction Scale–Short Version.

e NMP-Q: Nomophobia Questionnaire.

f DASS-21: Depression, Anxiety, and Stress Scale–21.

g ULS-8: University of California, Los Angeles, Loneliness Scale.

### Primary Intervention Effects (Group × Time Interaction)

Linear mixed model analyses revealed statistically significant group × time interaction effects across all primary outcomes (Table 2), consistently favoring the EG. Interaction effects were of moderate magnitude, including smartphone addiction (η_p_^2^=0.12; *P*=.004), nomophobia (η_p_^2^=0.10; *P*=.007), and global psychological distress (η_p_^2^=0.10; *P*=.009). Similar moderate interaction effects were observed for depression (η_p_^2^=0.09), anxiety (η_p_^2^=0.08), stress (η_p_^2^=0.08), and loneliness (η_p_^2^=0.08), indicating a consistent intervention-related improvement across behavioral and psychosocial domains.

**Table 2.** Linear mixed model results for intervention effects (group × time interaction) adjusted for age, sex, and BMI^a^.

Outcome	*F* test (*df*)	*P* value	η_p_^2^
SAS-SV^b^	8.9 (1, 67)	.004	0.12
NMP-Q^c^	7.8 (1, 67)	.007	0.10
DASS-21^d^ global	7.3 (1, 67)	.009	0.10
DASS-21 subscales			
Depression	6.9 (1, 67)	.01	0.09
Anxiety	6.2 (1, 67)	.02	0.08
Stress	5.9 (1, 67)	.02	0.08
ULS-8^e^	5.5 (1, 67)	.02	0.08

a *F* statistic represents fixed group × time interaction effects from linear mixed models with random intercepts for participants. Models were adjusted for age, sex, and BMI. Effect size (η_p_2) benchmarks: 0.01=small; 0.06=moderate; 0.14=large.

b SAS-SV: Smartphone Addiction Scale–Short Version.

c NMP-Q: Nomophobia Questionnaire.

d DASS-21: Depression, Anxiety, and Stress Scale–21.

e ULS-8: University of California, Los Angeles, Loneliness Scale.

### Pretest-Posttest Changes and Between-Group Differences

**Figure 2.**
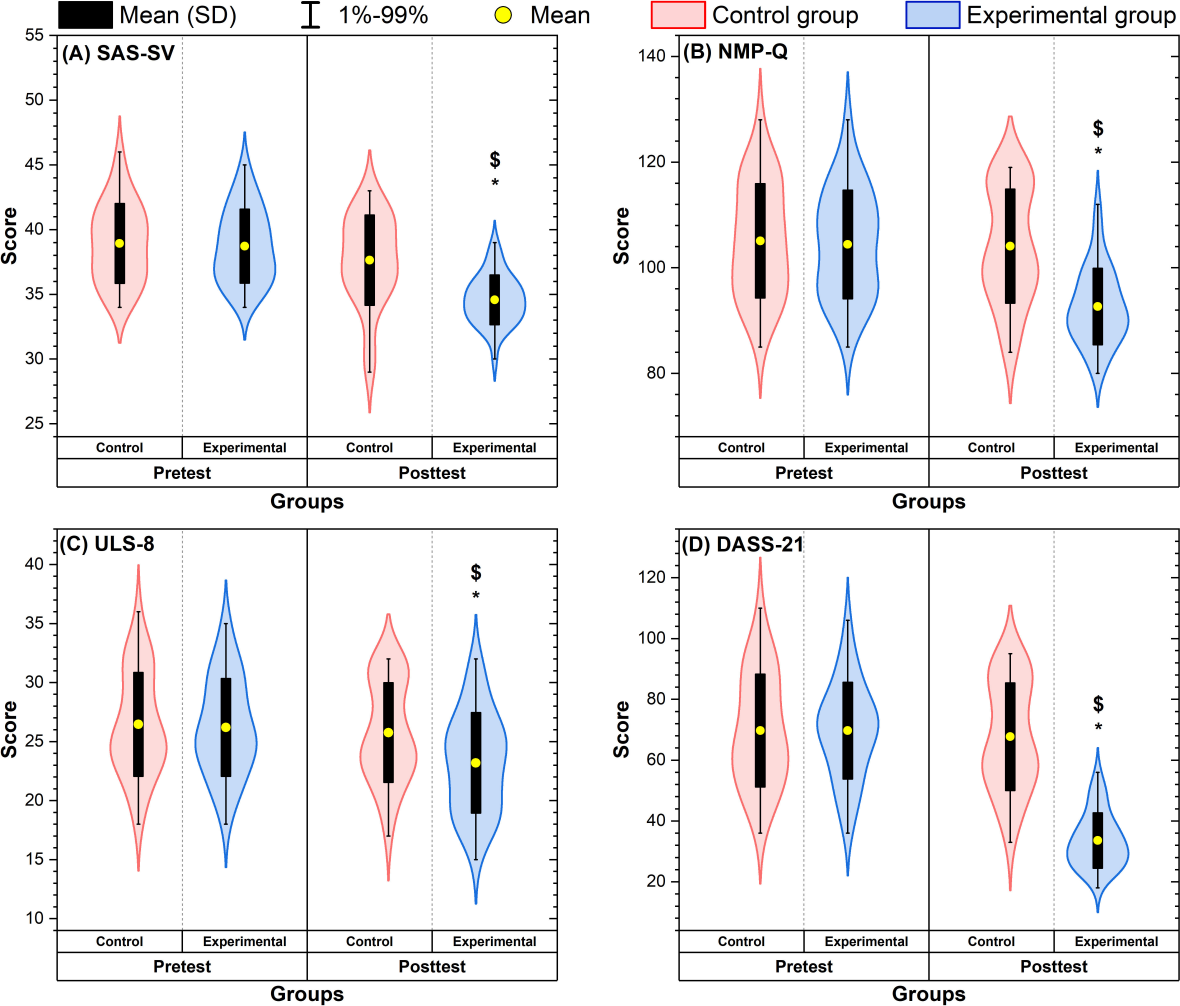
Effects of the 12-week intervention on smartphone addiction and psychological outcomes. Data are presented for (A) smartphone addiction (Smartphone Addiction Scale–Short Version; SAS-SV), (B) nomophobia (Nomophobia Questionnaire; NMP-Q), (C) loneliness (University of California, Los Angeles, Loneliness Scale; ULS-8), and (D) psychological distress (Depression, Anxiety, and Stress Scale–21; DASS-21) across the experimental and control groups. Violin plots show score distributions; yellow circles indicate means, and black bars indicate means and SDs. Whiskers represent the 1st to 99th percentiles. *Significant within-group change (paired *t* test) at *P*<.05. $Significant between-group difference at the posttest time point (independent *t* test) at *P*<.05.

Figure 2 illustrates the distribution of pre- and postintervention scores across outcome measures for the EG and CG. Visual inspection indicates clear reductions from before to after the intervention in the EG across smartphone addiction, nomophobia, loneliness, and psychological distress (global score and subscales), whereas score distributions in the CG remained largely stable over time. Exploratory within-group comparisons showed statistically significant pretest-posttest reductions in the EG across all outcomes (*P*<.05 in all cases), whereas no significant changes were observed in the CG (*P*>.05 in all cases). Between-group comparisons at the posttest time point favored the EG across all measures (*P*≤.02).

### Adjusted Postintervention Outcomes

After controlling for baseline scores, analyses of covariance confirmed significant postintervention group differences across all outcomes (Table 3). The EG demonstrated significantly lower adjusted posttest scores than the CG for smartphone addiction (η_p_^2^=0.12; *P*=.004), nomophobia (η_p_^2^=0.11; *P*=.006), global psychological distress (η_p_^2^=0.12; *P*=.005), depression (η_p_^2^=0.10; *P*=.008), anxiety (η_p_^2^=0.09; *P*=.01), stress (η_p_^2^=0.09; *P*=.02), and loneliness (η_p_^2^=0.09; *P*=.01). All effects were in the moderate range, supporting the robustness of the intervention after adjustment for baseline variability.

**Table 3.** Analysis of covariance (ANCOVA) results for posttest scores adjusted for baseline scores, age, sex, and BMI^a^.

Outcome	EG^b^, adjusted mean (SE)	CG^c^, adjusted mean (SE)	*F* test (*df*)	*P* value	η_p_^2^
SAS-SV^d^	34.6 (0.5)	37.8 (0.5)	9.1 (1, 66)	.004	0.12
NMP-Q^e^	95.2 (1.6)	101.7 (1.7)	8.2 (1, 66)	.006	0.11
DASS-21^f^ global	58.1 (1.7)	66.4 (1.8)	8.7 (1, 66)	.005	0.12
DASS-21 subscales					
Depression	23.6 (0.8)	27.1 (0.9)	7.4 (1, 66)	.008	0.10
Anxiety	18.8 (0.7)	21.6 (0.7)	6.8 (1, 66)	.01	0.09
Stress	16.3 (0.7)	19.1 (0.8)	6.2 (1, 66)	.02	0.09
ULS-8^g^	24.9 (0.4)	26.6 (0.4)	6.5 (1, 66)	.01	0.09

a ANCOVA models were adjusted for baseline (pretest) scores, age, sex, and BMI. Homogeneity of regression slopes was verified for all models. Effect size (η_p_2) benchmarks: 0.01=small; 0.06=moderate; 0.14=large. The Smartphone Addiction Scale–Short Version clinical cutoffs applied were ≥31 for boys and ≥33 for girls.

b EG: experimental group.

c CG: control group.

d SAS-SV: Smartphone Addiction Scale–Short Version.

e NMP-Q: Nomophobia Questionnaire.

f DASS-21: Depression, Anxiety, and Stress Scale–21.

g ULS-8: University of California, Los Angeles, Loneliness Scale.

### Mediation by Loneliness Reduction

**Figure 3.**
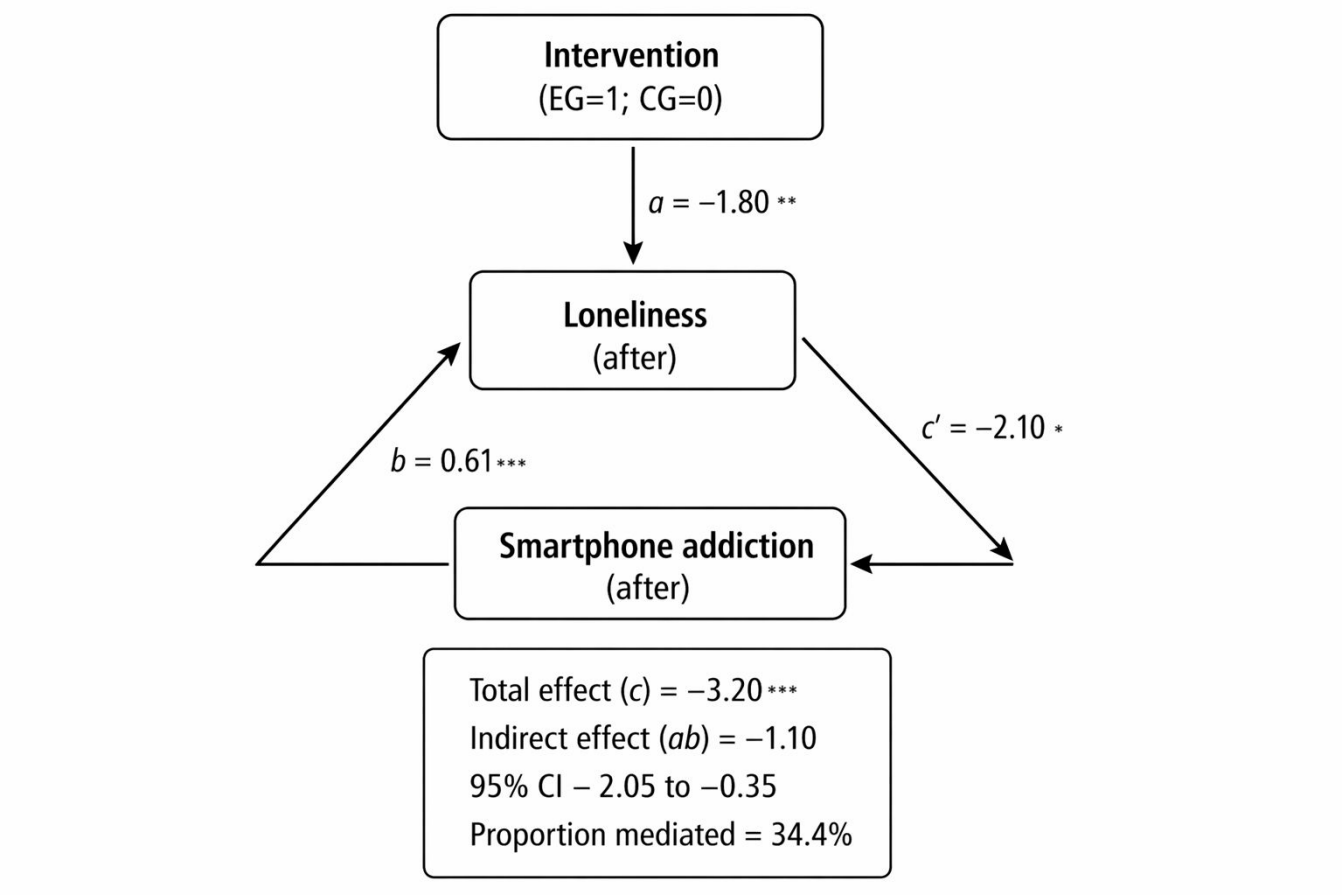
Loneliness (University of California, Los Angeles, Loneliness Scale) as a mediator of the intervention effect on smartphone addiction (Smartphone Addiction Scale–Short Version; SAS-SV)—simple mediation model (PROCESS Model 4) with 5000 bootstrap samples. The model was adjusted for age, sex, BMI, and baseline scores. The values shown are unstandardized coefficients. Path *a*=intervention→loneliness; path *b*=loneliness→SAS-SV. The indirect effect was significant (95% CI did not include 0), with 34.4% of the total effect mediated by loneliness. *P<.05; **P<.01; ***P<.001; CG: control group; EG: experimental group.

Mediation analysis showed that reductions in loneliness partially mediated the effect of the intervention on postintervention smartphone addiction (Table 4 and Figure 3). The indirect effect was statistically significant (*ab*=−1.10, 95% CI −2.05 to −0.35), accounting for 34.4% of the total effect. The intervention significantly reduced loneliness (*a*=−1.80; *P*=.002), and higher loneliness was associated with greater smartphone addiction (*b*=0.61; *P*<.001). The direct effect of the intervention remained significant after accounting for loneliness, indicating partial mediation. No adverse events or unintended effects were reported in either group during the trial period.

**Table 4.** Simple mediation analysis (PROCESS Model 4) adjusted for baseline scores, age, sex, and BMI^a^.

Path and effect	Unstandardized coefficient (SE; 95% CI)	*t* test (*df*)	*P* value
Total effect (*c*)			
^b^Effect of group on SAS-SV^b^ (after)	−3.20 (0.85; −4.89 to −1.51)	−3.76 (67)	<.001
Direct effect (*c*′)			
Effect of group on SAS-SV (after)	−2.10 (0.90; −3.89 to −0.31)	−2.33 (66)	.02
Indirect effect (combined; *ab*)	−1.10 (0.45; −2.05 to −0.35)	—^c^	—
Indirect effect (path specific)			
Effect of group on loneliness (*a*)	−1.80 (0.55; −2.88 to –0.72)	−3.27 (67)	.002
Effect of loneliness on SAS-SV (*b*)	0.61 (0.11; 0.39 to 0.83)	5.55 (66)	<.001

a Indirect effect 95% CIs were derived from 5000 bootstrap samples. The model was adjusted for age, sex, BMI, and baseline scores (Smartphone Addiction Scale–Short Version and University of California, Los Angeles, Loneliness Scale). Path *a*=intervention→loneliness; path *b*=loneliness→Smartphone Addiction Scale–Short Version. The proportion mediated was 34.4%.

b SAS-SV: Smartphone Addiction Scale–Short Version.

c Not applicable. The indirect effect (ab) is estimated using bootstrap procedures and therefore does not have a corresponding *t* value, df, or *P* value.

## Discussion

### Summary of Main Findings

This randomized controlled trial evaluated a 12-week, school-based intervention integrating traditional social sports games with mental exercises for Tunisian adolescents with elevated smartphone addiction. Linear mixed model analyses revealed significant group × time interaction effects of moderate magnitude (η_p_^2^=0.08-0.12), consistently favoring the EG over the CG. Participants receiving the TSSG-ME intervention demonstrated statistically significantly greater reductions in smartphone addiction, nomophobia, psychological distress (global and subscale scores), and loneliness. After adjusting for baseline scores, between-group differences at the posttest time point remained significant with moderate effect sizes. Furthermore, mediation analysis indicated that reductions in loneliness accounted for approximately 34.4% of the intervention’s total effect on smartphone addiction, supporting a partial mediation model.

### Interpretation and Comparison With the Literature

Reductions in smartphone addiction were among the most robust outcomes. These findings are consistent with those of prior studies showing that structured physical engagement and psychological support can reduce behavioral dependence on mobile technologies [38]. This aligns with meta-analytic findings that combined interventions integrating PA with psychological skill building produce more consistent reductions in smartphone addiction than single-component approaches [11,14]. This study contributes by demonstrating the efficacy of a culturally grounded program in a Tunisian context.

The term “traditional social sports games” denotes structured, rule-based PAs with inherent social organization and deep cultural resonance, distinguishing them from generic physical exercise [18,19]. The mental exercise component refers to a standardized protocol of evidence-based techniques adapted from sports psychology designed to build self-regulation and stress tolerance beyond simple relaxation [26,27]. The observed moderate effect sizes were clinically meaningful and consistent with those reported in other behavioral intervention trials targeting PSU [39]. They reflect a realistic and attainable impact, particularly given that our sample comprised adolescents with clinically elevated baseline symptoms who may have had greater room for improvement.

The reduction in nomophobia underscores the intervention’s potential to alleviate the anxiety associated with smartphone separation. By fostering rewarding, in-person social experiences and peer validation, the traditional games likely diminished the perceived indispensability of constant digital connectivity [6]. Concurrently, the mental exercise component equipped participants with concrete skills for managing emotional arousal, offering a healthier alternative to digital coping mechanisms [40].

Critically, our findings provide empirical support for the psychological mechanism proposed by SDT [15]. Reductions in loneliness mediated 34.4% of the intervention’s total effect on smartphone addiction, highlighting a key pathway. This suggests that the TSSG-ME program enhanced the basic psychological need for relatedness through prosocial, face-to-face interaction in traditional games, reducing perceived social isolation. Consequently, adolescents’ compensatory reliance on smartphones for social fulfillment likely decreased, alleviating addiction symptoms. This mechanism aligns with research identifying loneliness as a critical link between unmet social needs and PSU [41,42] and supports evidence that loneliness mediates the relationship between health-promoting behaviors and reduced maladaptive digital use [13,43]. Using culturally embedded games to foster relatedness also aligns with literature emphasizing their effectiveness in promoting sociability [44]. A key limitation is that mediation was tested with concurrent postintervention measures; temporal precedence cannot be fully established, so causal interpretation should be made cautiously.

The dual-component design posits complementary pathways: the traditional social sports games primarily targeted relatedness through social engagement and shared PA, whereas the mental exercise component targeted competence and autonomy through enhanced self-regulation [45]. Their integration likely addressed the multifaceted social-motivational and emotional-cognitive vulnerabilities underpinning smartphone addiction more comprehensively than either component alone [46]. Future research using factorial designs is necessary to disentangle their independent and synergistic contributions.

### Limitations

Several limitations should be acknowledged. First, the study relied on self-reported measures, which may be subject to response bias; future research should incorporate objective metrics such as device-based screen time tracking. Second, the culturally specific sample, while emphasizing the importance of contextual adaptation, may limit the generalizability of the findings to broader populations. Third, the absence of long-term follow-up assessments precludes conclusions regarding the sustainability of the intervention effects. Fourth, the use of a passive CG (standard physical education) rather than an active, time-matched comparator (eg, generic group sports or relaxation program) restricts the ability to attribute observed outcomes specifically to the traditional social games and mental training components as opposed to nonspecific factors such as increased social engagement or novelty. Finally, although effect sizes were statistically significant and clinically meaningful, they were moderate, highlighting the complex, multifactorial nature of PSU and suggesting the need for further optimization of the intervention.

### Conclusions

This study provides evidence that a culturally adapted, school-based intervention combining traditional social sports games and mental training can yield significant, moderate improvements in smartphone addiction, nomophobia, psychological distress, and loneliness among adolescents. The mediation of the treatment effect through reduced loneliness highlights the importance of enhancing real-world social connectedness, supporting a key mechanism derived from SDT. By providing alternative, healthier avenues for satisfying psychological needs for relatedness and competence, programs such as TSSG-ME may reduce reliance on maladaptive smartphone use.

Beyond individual outcomes, these findings suggest that integrating culturally relevant social activities into school-based programs may serve as a scalable strategy to support adolescent mental health in increasingly digital contexts. Policymakers and educators could leverage such approaches to mitigate the negative consequences of excessive smartphone use. Future research should use longitudinal designs, active CGs, and diverse populations to confirm causal pathways and assess long-term efficacy. However, given that mediation was examined cross-sectionally, causal interpretations should be made cautiously.

## Supplementary material

10.2196/85353 Checklist 1 CONSORT checklist.

## Abbreviations:

CG: control group
CONSORT: Consolidated Standards of Reporting Trials
DASS-21: Depression, Anxiety, and Stress Scale–21
EG: experimental group
NMP-Q: Nomophobia Questionnaire
PA: physical activity
PSU: problematic smartphone use
SAS-SV: Smartphone Addiction Scale–Short Version
SDT: self-determination theory
TSSG-ME: traditional social sports games with mental exercises
