# Supplementary material for: Traditional Social Sports Games and Mental Training for Smartphone Addiction and Psychological Distress in School-Aged Adolescents: Randomized Controlled Trial
Source: JMIR Mhealth Uhealth. 2026 May 4;14:e85353. doi: 10.2196/85353 (PMC13138712; doi:10.2196/85353)
Supplement: Checklist 1 [file mhealth-v14-e85353-s001.pdf]

Table 1. CONSORT 2017 Checklist for Randomized Trials Assessing Nonpharmacologic Treatments (NPTs)

Section/Topic	Item No.	CONSORT Item	Extension for NPT Trials	Location in Manuscript
Title and Abstract	1a	Identification as a randomized trial in the title		Title includes 'Randomized Controlled Trial' (p.1)
	1b	Structured summary of trial design, methods, results, and conclusions	Refer to CONSORT extension for abstracts for NPT trials	Abstract (all paragraphs) (p.2)
Introduction – **Background and Objectives**	2a	Scientific background and explanation of rationale		Introduction (paragraphs 1–6) (pp.3–4)
	2b	Specific objectives or hypotheses		End of Introduction (last paragraph) (p.4)
Methods – **Trial Design**	3a	Description of trial design (parallel), including allocation ratio	How care providers were allocated to groups	Two-arm parallel RCT; 1:1 allocation; instructors delivered both interventions (p.5)
	3b	Important changes after trial commencement		No changes (N/A)
Methods – **Participants**	4a	Eligibility criteria for participants	Eligibility of centers and care providers	Inclusion/exclusion criteria; 3 public middle schools; instructors described (pp.5–6, 8)
	4b	Settings and locations of data collection		Three public middle schools, Sfax region, Tunisia (p.5)
Methods – **Interventions**	5	Detailed description sufficient for replication	Precise details of experimental and comparator	Experimental and Control sections (pp.7–8)
	5a	Components and tailoring of interventions		TSSG (4 games, progressive complexity); ME (5 phases) (p.7)
	5b	Standardization of interventions		Standardized manual; adherence protocol (p.8)
	5c	Adherence of care		Training, supervision, manual (p.8)

		providers		
	5d	Adherence of participants		Attendance registers; 92% adherence (p.8)
Methods **– Outcomes**	6a	Primary and secondary outcomes, timing and assessment		SAS-SV; NMP-Q; DASS-21; UCLA-8; baseline & post (pp.8–10)
	6b	Changes to outcomes after commencement		No changes (N/A)
Methods **– Sample Size**	7a	How sample size was determined	Address clustering if applicable	G*Power (n=64 required; N=69 final); no clustering (pp.10–11)
	7b	Interim analyses and stopping guidelines		No interim analyses (N/A)
Randomization – **Sequence Generation**	8a	Method used to generate allocation sequence		Computer-generated random numbers (p.6)
	8b	Type of randomization and restrictions		Block randomization (block size=4) (p.6)
Randomization – **Allocation Concealment**	9	Mechanism to conceal allocation sequence		Sequentially numbered, opaque, sealed envelopes (p.6)
Randomization – **Implementation**	10	Who generated, enrolled, assigned participants		Independent researcher; lead author; research assistant (p.6)
Blinding	11a	Who was blinded and how	Whether co-intervention providers were blinded	Assessors and analysts blinded; participants/instructors not blinded (p.6)
	11b	Similarity of interventions (if relevant)		Not applicable
	11c	Attempts to limit bias if blinding not possible		Blinded assessors and analysts (p.6)
Statistical Methods	12a	Statistical methods for	Address clustering if applicable	Mixed models, ANCOVA, mediation; no clustering (p.11)

		primary/secondary outcomes		
	12b	Additional analyses (subgroup/adjusted)		ANCOVA and mediation (p.11)
Results – **Participant Flow**	13a	Numbers assigned, treated, analyzed	Number of centers/providers	EG=36; CG=33; 3 schools; 3 instructors; Figure 1
	13b	Losses and exclusions with reasons		Six excluded (EG=4; CG=2) (p.6)
	13c	Delay between randomization and intervention		Started within one week (p.7)
	New	Details of implementation fidelity		Attendance 92%; described (pp.7–8)
Results – **Recruitment**	14a	Recruitment and follow-up dates		Oct 2024 – Feb 2025 (p.5)
	14b	Why trial ended/stopped		Completed as planned
Results – **Baseline Data**	15	Baseline demographic and clinical characteristics	Description of providers and centers	Table 1; instructor qualifications; 3 schools
Results – **Numbers Analyzed**	16	Participants included in each analysis (ITT)		EG=36; CG=33; intention-to-treat
Results – **Outcomes and Estimation**	17a	Results, effect sizes, 95% CI		Tables 2–3 (η^2_p ; 95% CI)
	17b	Binary outcomes (if applicable)		No binary outcomes
Results – **Ancillary Analyses**	18	Other analyses (pre-specified vs exploratory)		Mediation (Table 4); ANCOVA
Results – **Harms**	19	Harms or unintended effects		No adverse events reported
Discussion – **Limitations**	20	Trial limitations and potential bias	Comparator, blinding, expertise	Self-report; single region; no long-term follow-up; passive control; no participant blinding
Discussion –	21	External validity	Intervention, participants,	Cultural specificity noted; school

**Generalizability**			centers	applicability discussed
Discussion – **Interpretation**	22	Interpretation consistent with results		Discussed within theoretical framework
Other Information – **Registration**	23	Registration number and registry		PACTR202601838702413 (p.5)
Other Information – **Protocol**	24	Access to full protocol		Available upon request
Other Information – Funding	25	Sources of funding and role of funders		Funding section: "Funded by Princess Nourah bint Abdulrahman University Researchers Supporting Project (PNURSP2026R145). The funders had no role in study design, data collection, analysis, interpretation, manuscript preparation, or decision to publish." (After Discussion)
